# Discrepancy of p16 immunohistochemical expression and HPV RNA in penile cancer. A multiplex in situ hybridization/immunohistochemistry approach study

**DOI:** 10.1186/s13027-021-00361-8

**Published:** 2021-03-31

**Authors:** Federico Zito Marino, Rosalaura Sabetta, Francesca Pagliuca, Matteo Brunelli, Gabriella Aquino, Sisto Perdonà, Gerardo Botti, Gaetano Facchini, Francesco Fiorentino, Giovanni Di Lauro, Marco De Sio, Ferdinando De Vita, Giorgio Toni, Rodolfo Borges Dos Reis, Luciano Neder, Renato Franco

**Affiliations:** 1 Pathology Unit, Department of Mental and Physical Health and Preventive Medicine, University of Campania “L. Vanvitelli”, Complesso di Santa Patrizia, Via Luciano Armanni, 5, 80138 Naples, Italy; 2 Department of Pathology, University of Verona, Verona, Italy; 3 Pathology Unit, Istituto Nazionale Tumori, Fondazione G. Pascale, IRCCS, 80131 Naples, Italy; 4 Department of Urology, Istituto Nazionale Tumori, Fondazione G. Pascale, IRCCS, 80131 Naples, Italy; 5 Medical Oncology Unit, S.M. delle Grazie Hospital, Via Domitiana, 80078 Pozzuoli, NA Italy; 6 Pathology Unit, S.M. delle Grazie Hospital, Via Domitiana, 80078 Pozzuoli, NA Italy; 7 Urology Unit, S.M. delle Grazie Hospital, Via Domitiana, 80078 Pozzuoli (NA), Italy; 8 Urology Unit, Department of Woman, Child and General and Specialized Surgery, University of Campania ’Luigi Vanvitelli, 80138 Naples, Italy; 9 Division of Medical Oncology, Department of Precision Medicine, School of Medicine, “Luigi Vanvitelli” University of Campania, Naples, Italy; 10 Laboratoire Central d’Anatomie pathologique, Hôpital universitaire de Nice, Université Côte d’Azur, 06000 Nice, France; 11 Department of Surgery and Anatomy, Urology Division, Ribeirao Preto School Medicine, University of São Paulo, 14049 900 Ribeirao Preto, Brazil; 12 Department of Pathology and Forensic Medicine, Ribeirão Preto Medical School, University of São Paulo, 14049 900 Ribeirão Preto, SP Brazil; 13 Molecular Oncology Research Center, Barretos Cancer Hospital, 14784400 Barretos, SP Brazil

**Keywords:** ISH, HPV, p16, Penile carcinoma, Multiplex HPV RNA ISH /p16 IHC

## Abstract

**Background:**

The high-risk human papillomavirus (HPV) infection represents one of the main etiologic pathways of penile carcinogenesis in approximately 30–50 % of cases. Several techniques for the detection of HPV are currently available including Polymerase chain reaction-based techniques, DNA and RNA in situ hybridization (ISH), p16 immunohistochemistry (IHC). The multiplex HPV RNA ISH/p16 IHC is a novel technique for the simultaneous detection of HPV E6/E7 transcripts and p16INK4a overexpression on the same slide in a single assay. The main aim of this study was to evaluate the discrepancy of p16 IHC expression relatively to HPV RNA ISH in penile cancer tissue.

**Methods:**

We collected a series of 60 PCs. HPV has been analysed through the RNA ISH, p16 IHC and the multiplex HPV RNA ISH/p16 IHC.

**Results:**

The multiplex HPV RNA ISH /p16 IHC results in the series were in complete agreement with the previous results obtained through the classic p16 IHC and HPV RNA scope carried out on two different slides. The multiplex HPV RNA ISH /p16 IHC showed that HPV positivity in our series is more frequently in usual squamous cell carcinoma than in special histotypes (19 out of 60 − 15 %- versus 6 out of 60 − 10 %-), in high-grade than in moderate/low grade carcinomas (6 out of 60 − 10 %- versus 4 out of 60 − 6.7 %-). In addition, our data revealed that in 5 out of 20 cases with p16 high intensity expression is not associated with HPV RNA ISH positivity.

**Conclusions:**

Our findings emphasize that the use of p16 as a surrogate of HPV positivity was unsuccessful in approximatively 8 % of cases analysed in our series. Indeed, p16 IHC showed a sensitivity of 100 % and a specificity of 71 %, with a positive predictive value (PPV) of 54 % and a negative predictive value of 100 %; when considering high intensity, p16 IHC showed a sensitivity of 100 %, a specificity of 89 %, with a PPV of 75 % and NPV of 100 %.

Since HPV positivity could represent a relevant prognostic and predictive value, the correct characterization offered by this approach appears to be of paramount importance.

## Background

Penile carcinoma (PC) is a rare and aggressive disease with an incidence of 0.4 % in the United States and Western Europe, while the incidence is higher in less developed countries, including Africa, Asia and South America, estimated around 6.0 % [1–3]. The most frequent histotype is the conventional squamous cell carcinoma with or without keratinization, accounting for approximatively 70–75 % of cases, followed by basaloid, sarcomatoid and warty subtypes. The verrucous and condylomatous subtypes are rare histotype with a much better prognosis [4].

The PC development can be attributed to two different pathogenic pathways, the chronic inflammation of the lichen sclerosus being the most frequent ; then the infection by the high-risk human papilloma virus (HPV) is relatively less common [2, 4–6]. Indeed, the frequency of HPV related PC ranges from 30 to 50 %. Genotypes more frequently detected are 16, 18, 31, 33, 45, 56, and 65 [4, 7–10]. HPV-related PC constitutes a specific subset with a good prognosis, since the 5year diseasespecific survival of such patients is better than patients with HPVurelated PC (93 % vs. 78 %) [11, 12].

The new World Health Organization (WHO) attributes a great importance to HPV-related cancer, revising the previous classification of PCs based exclusively on the morphology. Thus, PCs are subtyped the into HPV-related and HPV-unrelated tumors [13, 14].

The main HPV-related subtypes are frequently basaloid and warty carcinomas, while the conventional squamous cell carcinoma, the papillary and the verrucous carcinomas are mainly non-HPV-related [4, 13, 15].

The HPV can be detected through direct and indirect different assays, including the polymerase chain reaction (PCR), p16 immunohistochemistry (IHC) and in situ hybridization (ISH) analysis. PCR assay detects HPV DNA identifying both transient and persistent infections. This method is a highly sensitive and it allows the identification of the different genotypes of the virus [16, 17]. ISH analysis allows HPV detection preserving the morphological context, allowing the HPV locations in the tumor tissue. Several studies reported high sensitivity and specificity of DNA ISH in PC, whereas RNA ISH is not frequently used to detect HPV in such cancer [18–20].

IHC represents a surrogate method to detect HPV infectious, since it is based on the identification of the p16 expression, being considered as a marker for high-risk HPV-induced transformation. Indeed, since p16 is accumulated as an effect of HPV E7 oncoprotein, its overexpression seems to be a critical early event in carcinogenesis [21]. Thus, it is commonly used in the clinical practice, also in the diagnosis of HPV-related cancers in other anatomic sites. Although p16 IHC expression has been established as a highly valid surrogate marker, previous data showed a disagreement between HPV infectious detected through molecular approach and p16 IHC expression [22–24]. Indeed, as previously reported in other HPV-related cancer types, such as cervical cancers and oropharyngeal squamous cell carcinoma (OPSCC), RNA ISH could be a useful tool to identify HPV infectious in all cases where p16 IHC or DNA ISH test are negative or equivocal for [25, 26]. We previously developed a new multiplex approach to identify HPV infectious carrying out HPV RNA ISH and p16 IHC on the same slide to simultaneously detect HPV E6/E7 transcripts and p16INK4a overexpression [25]. We validated this novel assay in a histopathological series including cervical cancers and OPSCCs [25]. This multiplex approach could improve the detection of HPV in PCs, contributing to a better classification of HPV-related PCs. In this view, we have analysed the HPV in a multicentric series of PCs using the multiplex HPV RNA ISH /p16 IHC assay.

## Methods

### Specimens

A series of 60 penile carcinomas were included in our study. All cases were collected in our records at the University of Campania “L. Vanvitelli” Hospital, the Istituto Nazionale Tumori, Fondazione G. Pascale, the S.M. delle Grazie Hospital and the University Ribeirão Preto Hospital of São Paulo,. The series included surgical samples and wide biopsies, formalin-fixed paraffin-embedded (FFPE) samples. Sections of 4 μm thickness from each block with a mean of 3 blocks per tumor) were stained with hematoxylin-eosin. All cases were reviewed according to the WHO histopathological classification [13, 14].

### p16 immunohistochemistry

p16 IHC was carried out with a proprietary kit CIN tec Histology; MTM laboratories AG) using the clone E6H4 on a Ventana Benchmark autostainer (Ventana Medical Systems, Tucson, AZ, USA) for the detection of p16INK4a antigen. A cervical carcinoma with high p16 expression was used as a positive control. The primary antibody was omitted from negative controls.

In our analysis we identified the subgroups with different p16 IHC staining, as follows: p16 high expression: tumors with staining ≥ 70 % nuclear and cytoplasmic staining; p16 moderate expression: tumors with staining 30–70 % nuclear and cytoplasmic staining; p16 low expression: tumors with staining 10–30 % nuclear and cytoplasmic staining; p16 negative: tumors with staining 1–10 % nuclear and cytoplasmic staining. In addition, also intensity was evaluated as previously reported [24]. The slides were independently evaluated by three separate observers.

### Automated HPV RNA in situ hybridization

Section 4 $\mu$m of each case are used to perform HPV RNA ISH test. Detection of high-risk-HPV E6/E7 mRNA was performed using Ready-to-use reagents from RNAscope 2.5 LS Reagent Kit-BROWN and the HPV-HR18 probe cocktail (Advanced Cell Diagnostics) that were loaded onto the Leica Biosystems’ BOND RX Research Advanced Staining System according to the user manual (Doc. No. 322,100-USM). The slides were independently evaluated by three separate observers. Ubiquitin C and dapB were used as positive and negative controls, respectively. A positive HPV ISH test result was defined as positive if any of the malignant cells showed brown punctate dot-like nuclear and/or cytoplasmatic positivity [27].

### Multiplex HPV RNA in situ hybridization/p16 immunohistochemistry

We performed the multiplex HPV RNA ISH /p16 IHC assay according to the protocol previous reported [25].

The protocol utilizes the Diaminobenzidine (DAB) chromogen of the Bond Polymer Refine kit to staining HPV E6/E7 mRNA, the Fast Red chromogen of the Bond Polymer Red Refine kit to staining p16 and hematoxylin to counterstain. Detection of high-risk-HPV E6/E7 mRNA was performed using ready-to-use reagents from RNAscope® 2.5 LS Reagent Kit-BROWN and the HPV-HR18 probe cocktail (Advanced Cell Diagnostics) that were loaded onto the Leica Biosystems’ BOND RX Research Advanced Staining System according to the user manual (Doc. No. 322,100-USM). The target-specific probes include the E6 and E7 mRNA of 18 h-HPV genotypes HPV (16,18, 26,31, 33, 35, 39, 45, 51, 52, 53, 56, 58, 59, 66, 68, 73 and 82). The Ubiquitin C a constitutively expressed endogenous gene was used as positive control to assess the presence adequate RNA quality and avoid a false-negative result. The dapB test was used as negative control to assess non-specific staining, for a comparison in the cases with negative or weakly stained HPV staining.

In brief, 4 μm sections were baked and deparaffinized on the instrument, followed by epitope retrieval using Leica Epitope Retrieval Buffer 2 at 95 °C or at 88 °C for 15 min and protease treatment 15 min at 40 °C. Probe hybridization, signal amplification trough different AMP reagent AMP 1–6) and colorimetric detection were subsequently performed. Several washes were performed, subsequently the ready-to-use primary antibody clone E6H4 for the detection of p16INK4a antigen was incubated and colorimetric detection was performed. Finally, a hematoxylin staining was carried out.

When the run is completed and the slide trays are removed, the covertiles are carefully lifted upward by the neck to remove. The slides are dehydrated through 2 changes each of 70 %, 95 %, and 100 % alcohol and 2 changes of xylene, before coverslipping. A positive HPV ISH test result was defined as positive if any of the malignant cells showed brown punctate dot-like nuclear and/or cytoplasmatic positivity. p16 IHC was positive if nuclear and cytoplasmic red staining was observed according to the above score. The slides were independently analyzed by three separate observers evaluating simultaneously HPV mRNA and p16 expression.

## Results

### Clinic-pathological features

In our series, patients older than 60 years were 28; the tumor site was the glans in 54 cases, the foreskin in 3 and the shaft in 3. Usual squamous cell carcinoma (USCC) was observed in 49, whilest a special histotype in 11 (papillary in 2 cases, verrucous in 3 cases, basaloid in 4 cases, warty in 1 case and mixed warty-basaloid in 1 case). In USCC histotype, the low grade (G1) was recorded in 15 cases, the moderate grade (G2) in 22, the high grade (G3) in 13. The grade was not applicable in 10 cases since they were special types and in situ carcinomas. Finally, the tumors were staged T1 in 19 cases, T2 in 17, T3 in 11 and T4 in 3, two cases were in situ carcinoma and the stage was not available in 8 cases. The data were reported in Table 1.

**Table 1:** Clinical and pathological features of penile carcinomas in our series

		*P16 IHC*		*HPV ISH*		multiplex HPV RNA ISH /p16 IHC			
	***Total***	**Positive** **> 70 % N. (%)**	**Negative** **N. (%)**	**Positive** **N. (%)**	**Negative** **N. (%)**	**P16+** **ISH+** **N. (%)**	**P16+** **ISH-** **N. (%)**	**P16-** **ISH-** **N. (%)**	**P16-** **ISH+** **N. (%)**
***Mean age (years)***									
< 60	28 (46.7 %)	14 (23.3 %)	14 (23.3 %)	6 (10 %)	22 (36.7 %)	6 (10 %)	8 (13.3 %)	14 (23.3 %)	
≥ 60	32 (53.3 %)	14 (23.3 %)	18 (30 %)	9 (15 %)	23 (38.3 %)	9 (15 %)	5 (8.3 %)	18 (30 %)	
***Anatomical Location***									
glans	54 (90 %)	25 (46.3 %)	29 (53.7 %)	13 (24.1 %)	41 (75.9 %)	13 (24.1 %)	12 (22.2 %)	29 (53.7 %)	
foreskin	3 (5 %)	1 (1.7 %)	2 (3.3 %)	1 (1.7 %)	2 (3.3 %)	1 (1.7 %)		2 (3.3 %)	
shaft	3 (5 %)	2 (3.3 %)	1 (1.7 %)	1 (1.7 %)	2 (3.3 %)	1 (1.7 %)	1 (1.7 %)	1 (1.7 %)	
***T stage***									
T1	19 (31.7 %)	9 (15 %)	10 (16.7 %)	4 (6.7 %)	15 (25 %)	4 (6.7 %)	5 (8.3 %)	10 (16.7 %)	
T2	17 (28.3 %)	5 (8.3 %)	12 (20 %)	1 (1.7 %)	16 (26.7 %)	1 (1.7 %)	4 (6.7 %)	12 (20 %)	
T3	11 (18.3 %)	6 (10 %)	5 (8.3 %)	4 (6.7 %)	7 (11.7 %)	4 (6.7 %)	2 (3.3 %)	5 (8.3 %)	
T4	3 (5 %)	1 (1.7 %)	2 (3.3 %)	1 (1.7 %)	2 (3.3 %)	1 (1.7 %)		2 (3.3 %)	
in situ	2 (3.3 %)	2 (3.3 %)		2 (3.3 %)		2 (3.3 %)			
NA	8 (13.3 %)	5 (8.3 %)	3 (5 %)	3 (5 %)	5 (8.3 %)	3 (5 %)	2 (3.3 %)	3 (5 %)	
***Histological classification***									
USCC	49 (81.7 %)	19 (31.7 %)	30 (50 %)	9 (15 %)	40 (66.7 %)	9 (15 %)	11 (18.3 %)	29 (48.3 %)	
papillary type	2 (3.3 %)	2 (3.3 %)		1 (1.7 %)	1 (1.7 %)	1 (1.7 %)		1 (1.7 %)	
verrucous	3 (5 %)	1 (1.7 %)	2 (3.3 %)	1 (1.7 %)	2 (3.3 %)	1 (1.7 %)		2 (3.3 %)	
basaloid	4 (6.7 %)	4 (6.7 %)		2 (3.3 %)	2 (3.3 %)	2 (3.3 %)	2 (3.3 %)		
warty	1 (1.7 %)	1 (1.7 %)		1 (1.7 %)		1 (1.7 %)			
mixed (warty-basaloid)	1 (1.7 %)	1 (1.7 %)		1 (1.7 %)		1 (1.7 %)			
***Histological grade***									
G1	15 (25 %)	7 (11.7 %)	8 (13.3 %)	2 (3.3 %)	13 (21.7 %)	2 (3.3 %)	5 (8.3 %)	8 (13.3 %)	
G2	22 (36.7 %)	7 (11.7 %)	15 (25 %)	2 (3.3 %)	20 (33.3 %)	2 (3.3 %)	5 (8.3 %)	15 (25 %)	
G3	13 (21.7 %)	7 (11.7 %)	6 (10 %)	6 (10 %)	7 (11.7 %)	6 (10 %)	1 (1.7 %)	6 (10 %)	
NAP (special types and in situ carcinomas)	10 (16.7 %)	7 (11.7 %)	3 (5 %)	5 (8.3 %)	5 (8.3 %)	5 (8.3 %)	2 (3.3 %)	3 (5 %)	
***Total***	60 (100 %)	28 (46.7 %)	32 (53.3 %)	15 (25 %)	45 (75 %)	15 (25 %)	13 (21.7 %)	32 ( 53.3 %)	0

*USCC* usual squamous cell carcinoma; *NA* not available; *NAP* not applicable

### P16 immunohistochemisty and HPV ISH

P16 overexpression was recorded in 28 cases, 19 in USCC; 9 in special histotypes, p16 overexpression was equally distributed in older and younger patients. Particularly, p16 low intensity expression was observed in 5 cases (8.3 %), moderate in 3 cases (5 %) and high intensity in 20 cases (33.3 %).

HPV-ISH was observed in 15 cases, 9 in USCC; 6 in special histotypes, HPV-ISH positivity was more frequent in older patients, being observed in 9 cases older than 60 yrs.

**Fig. 1:**
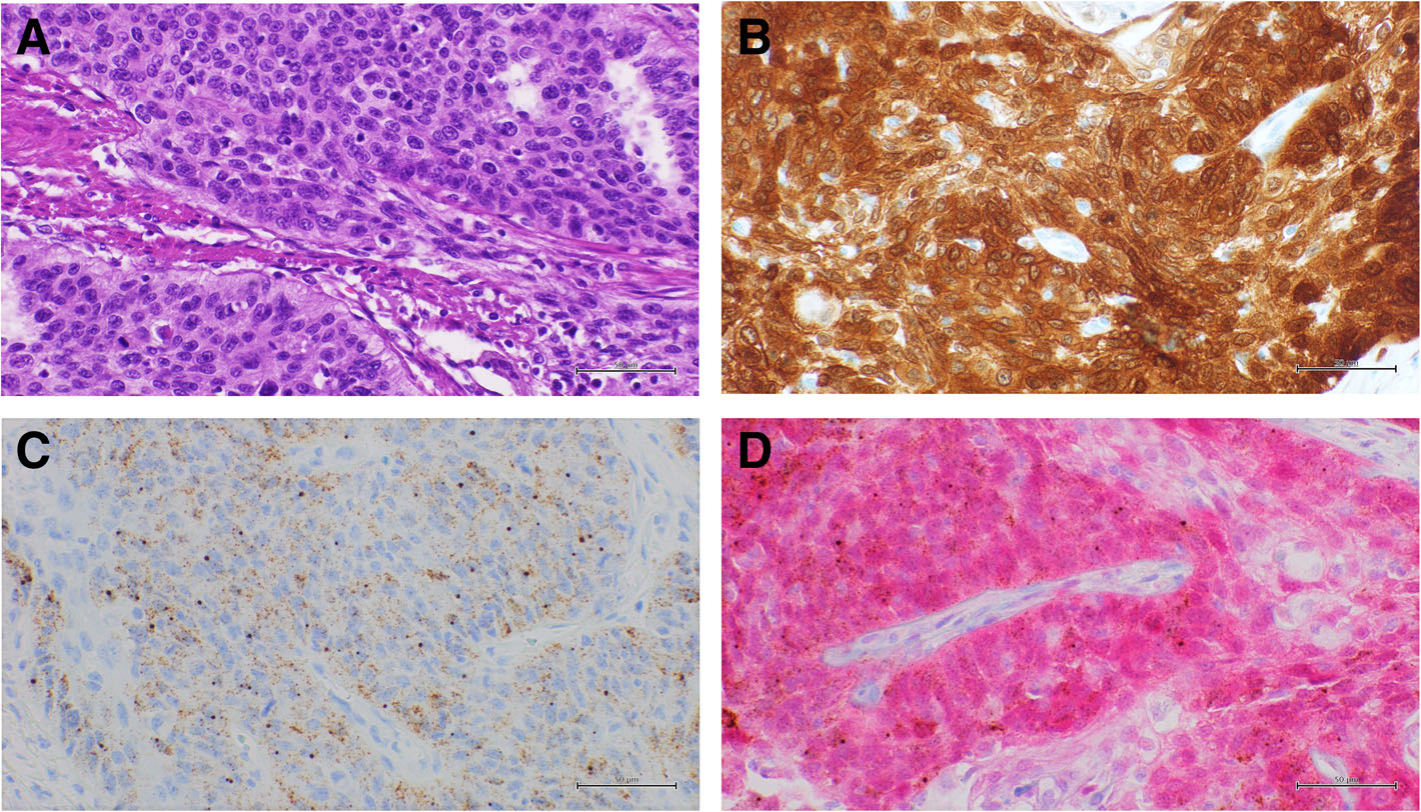
Representative results of a basaloid penile carcinoma with high p16 IHC expression and HPV RNA expression.** a**: Hematoxylin and Eosin (H & E) staining (40x); **b**: positive p16 IHC, DAB staining (40x); **c**: positive HPV RNA in situ hybridization, DAB staining (40x); **d**: multiplex HPV RNA ISH/p16 IHC: positive p16 IHC Fast Red staining and positive HPV RNA ISH DAB staining (40x)

**Fig. 2:**
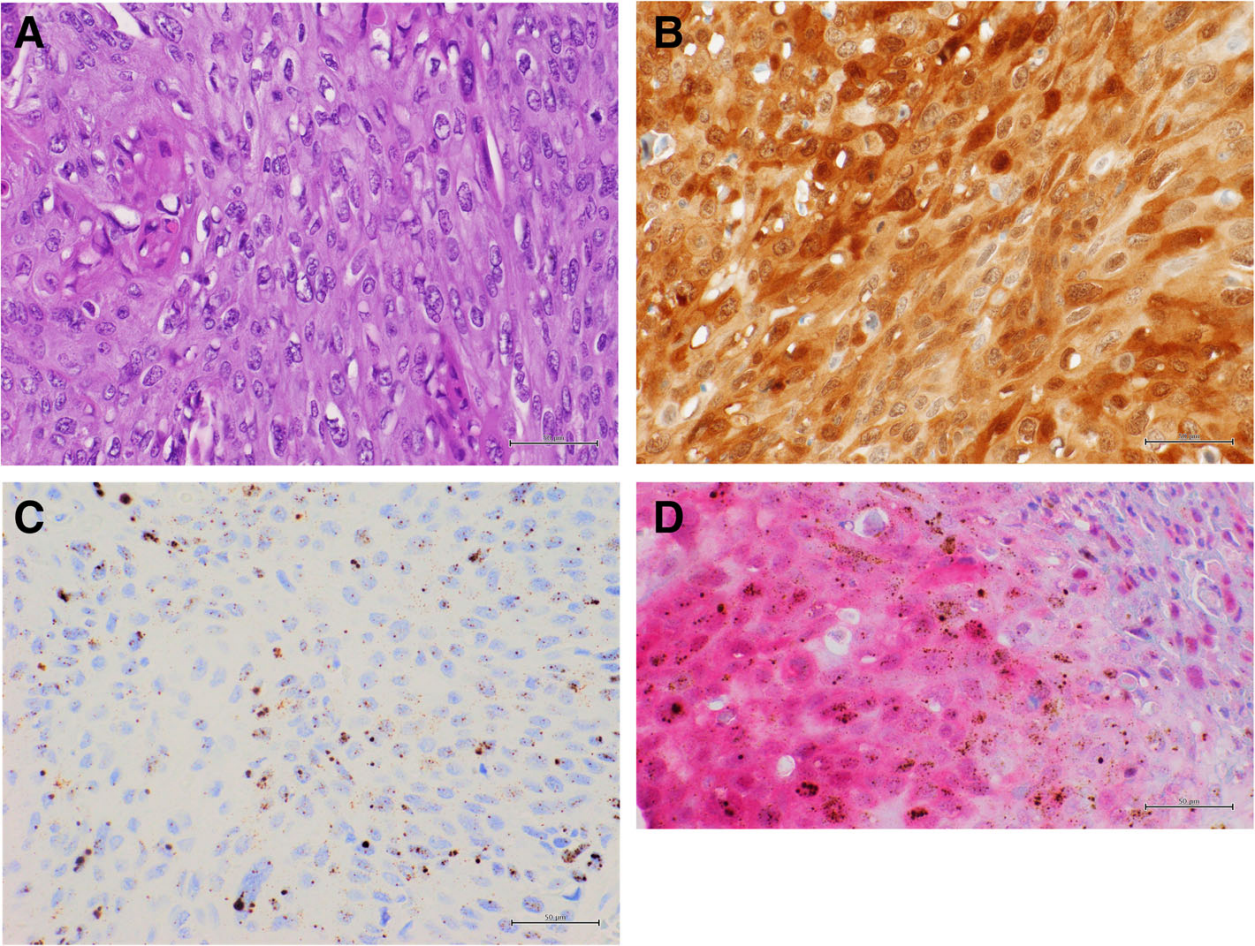
Representative results of a warty penile carcinoma with high p16 IHC expression and HPV RNA expression. **a**: Hematoxylin and Eosin (H & E) staining (40x); **b**: positive p16 IHC, DAB staining (40x); **c**: positive HPV RNA in situ hybridization, DAB staining (40x); **d**: multiplex HPV RNA ISH/p16 IHC: positive p16 IHC Fast Red staining and positive HPV RNA ISH DAB staining (40x)

**Fig. 3:**
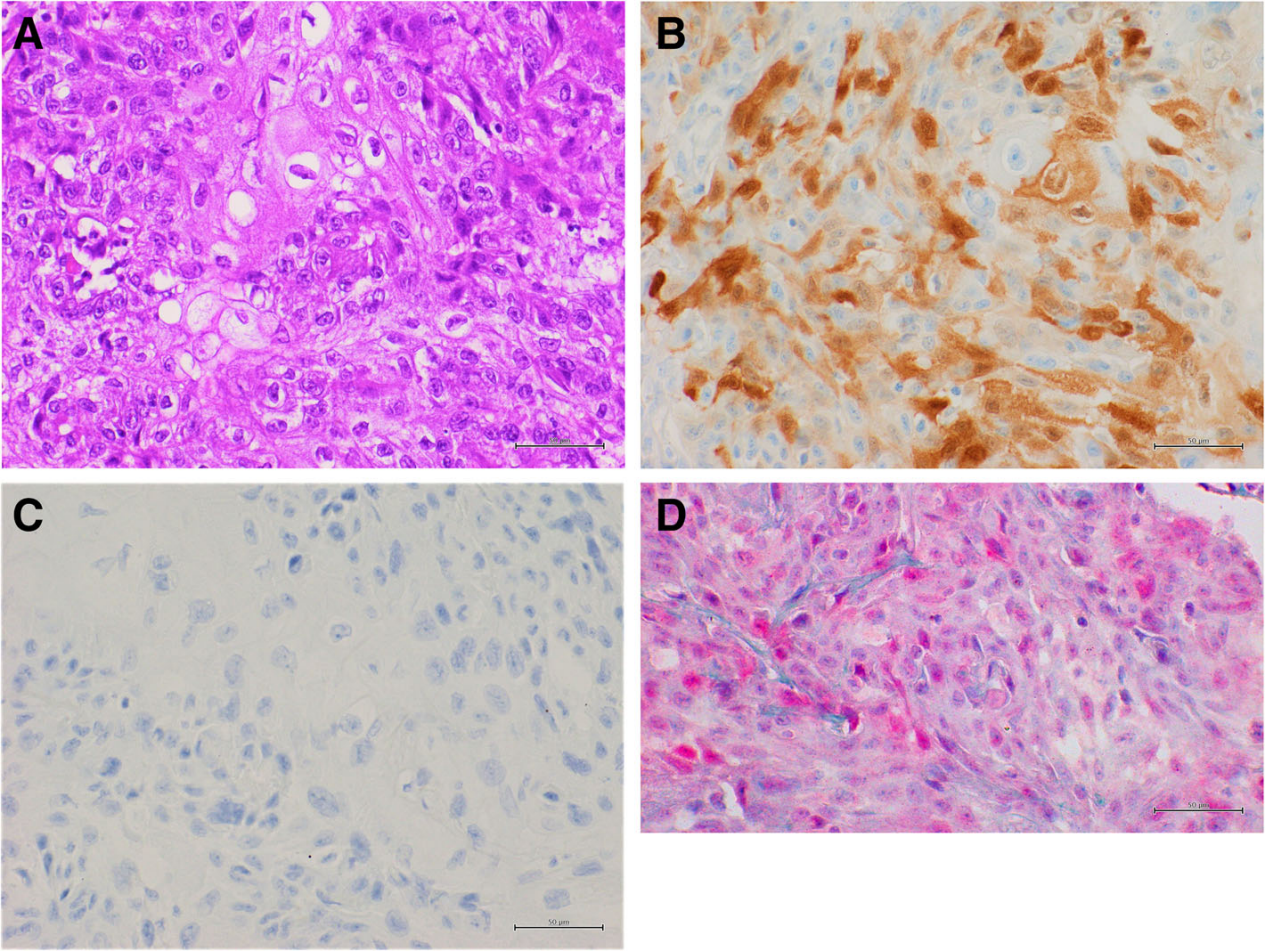
Representative results of a usual squamous cell penile carcinoma with high p16 and HPV RNA ISH negative.** a**: Hematoxylin and Eosin (H & E) staining (40x); **b**: positive p16 IHC, DAB staining (40x); **c**: negative HPV RNA in situ hybridization, DAB staining (40x); **d**: multiplex HPV RNA ISH/p16 IHC: positive p16 IHC Fast Red staining and negative HPV RNA ISH DAB staining (40x)

The data of multiplex approach were fully concordant with single approach methods, also considering the cut-off values and the intensity of p16 overexpression. Notably, all HPV-ISH positive cases overexpress p16 (Figs. 1 and 2); while p16 overexpression (5 cases with low intensity; 3 with moderate intensity and 5 with high intensity) was also observed in 13 HPV-ISH negative cases (Table 2) (Fig. 3).

**Table 2:** Correspondence between p16 immunohistochemical expression and the HPV RNA ISH in our series

*P 16 IHC*	*HPV ISH*	
	***N. Pos (%)***	***N. Neg(%)***
***negative***	0 (0 %)	32 (53,3 %)
***low***	0 (0 %)	5 (8.3 %)
***moderate***	0 (0 %)	3 (5 %)
***high***	15 (25 %)	5 (8.3 %)
***Total***	15 (25 %)	45 (75 %)

Thus, when considering only the cut-off without the intensity, p16 IHC showed a sensitivity of 100 % and a specificity of 71 %, with a positive predictive value (PPV) of 54 % and a negative predictive value of 100 %; when considering cut-off combined to high intensity, p16 IHC showed a sensitivity of 100 %, a specificity of 89 %, with a PPV of 75 % and NPV of 100 % (Table 3).

**Table 3:** Specificity and Sensitivity of p16 IHC in our series

	P16 IHC low/moderate/high	P16 IHC high
**specificity**	71 %	89 %
**sensitivity**	100 %	100 %
**PPV**	54 %	75 %
**NPV**	100 %	100 %

*IHC* immunohistochemistry; *PPV* positive predictive value; *NPV* negative predictive value

## Discussion

The role of HPV is well documented in the pathogenesis of several human cancer types, including cervical, oropharyngeal, penile, vaginal, vulvar and anal cancers [28]. Indeed, HPV is associated with the development of approximately 90 % of cervical carcinomas and the prophylactic vaccination is recommended in girls in order to prevent the infection [18]. In contrast, there is no recommendation for the use of the HPV prophylactic vaccination in boys due to the differences in the risk patterns for HPV-related PC [16, 17].

Although the frequency of the HPV in PC is lower than in cervical cancer, the HPV-related PCs constitute a subgroup with distinct biology and generally associated with a better prognosis and good response to the treatment than HPV-unrelated cancer. The new WHO classification has well identified a subset of PCs whose etiopathogenesis is closely associated with HPV infection. The accurate identification of the HPV-related PCs is necessary for the appropriate classification and management of these tumors.

Previous findings in PC demonstrated the overall correspondence of p16 IHC overexpression and the HPV presence; a strong association (*p* < 0.000001) was reported between HR-HPV and p16 showing entire and continuous cytoplasmic or nuclear staining in all neoplastic cells, except in the hyperkeratotic or parakeratotic areas when present [8]. Although the strong association with the molecular assay, p16 IHC shows a sensitivity of 67 % (95 % CI: 57-75 %) and a specificity of 91 % (95 % CI: 88-95 %) for the identification of HPV infection, suggesting a limited diagnostic capability of such test in detecting HPV [8].

Martins and colleagues showed that up to 50 % of PC cases positive to HPV molecular assay were negative for p16 IHC expression; in addition, 21 out of 22 cases positive for p16 IHC were confirmed for the presence of HPV [29]. Similarly do Carmo Alves Martins et al. demontrated that the p16 overexpression was observed exclusively in 12 out of 26 HPV PCR-positive cases with 8 out of 12 HPV-16 positive cases. Furthermore, 4 HPV PCR-negative cases showed p16 overexpression [30].

The discordance between HPV PCR assay and p16 expression could be explained by biological reasons, including a non-oncogenic or transient HPV infection, an inactivation of *CDNK2A*, the gene encoding for p16, due to the loss of heterozygosity or the promoter hypermethylation [8, 24].

Beyond biological issues, the p16 IHC positivity in cases defined as HPV negative through PCR methods could be attributed to a detection failure associated with the technical limits, such as the DNA degradation [8].

Although the PCR assay currently represents the gold standard for the HPV detection and genotyping, this method is mainly indicated in fresh-frozen samples while it has a limited yield in FFPE [25, 31].

The RNA ISH is an innovative tool to detect the HPV E6/E7 mRNA transcripts on FFPE samples, providing information not only related to the presence of the virus but also the transcriptionally-active status [32]. The RNA ISH has significantly improved the detection of HR-HPV in OPSCC, leading to an accurate diagnosis of misclassified cases through p16 IHC [26].

In OPSCC, the combination of various assays to detect HPV improves the accuracy compared to the use of a single method, thus diagnostic algorithm including p16 IHC and molecular approaches is used in the clinical practice [25]. The discordance between p16 expression and ISH techniques used for HPV detection has been previously reported, particularly, using HPV ISH as a gold standard in oropharyngeal SCC, p16 IHC expression for HPV detection has been reported to have a variable sensitivity and specificity, ranging from 53 to 100 % and from 54 to 100 % respectively [33]. Both sensitivity and specificity are better when considering RNA ISH as the gold standard rather than DNA ISH [33]. Thus, p16 might be an optimal surrogate, but it is expressed in other HPV-unrelated tumors. Further ambiguity of p16 overexpression is related to the used cut-off > 70 % and the intensity of the tumor cells [33]. Not extensive experiences of HPV detection in PCs are reported. Particularly, extraction methods for HPV DNA detection revealed a concordance of HPV positive sample with p16 overexpression from 83.1 to 85.3 % of cases [6, 15]. Rare series reported the comparison of HPV-ISH and p16 overexpression, underlying HPV RNA-ISH and p16 overexpression discordance, with both more 16 overexpressing tumors respect to HPV-ISH positivity cases and *vice versa* [34, 35].

Our data showed 13 discordant cases between HPV RNA ISH and p16 IHC results, considering only the cut-off without the intensity of p16 expression. However, when considering the cut-off combined to the expression intensity, there were, the 5 discordant cases. Particularly, when considering only the cut off of 70 % without the intensity of p16 expression, the concordance was recorded in 78.3 % of cases, p16 being more frequently positive, with a sensitivity of 100 %, a specificity of 71 %, a PPV of 54 % and a NPV of 100 %. Furthermore, considering a combined score including the cut off of 70 % and the high intensity of p16 IHC expression, the concordance was recorded in 91.7 % of cases, with a sensitivity of 100 %, a specificity of 89 %, a PPV of 75 % and a NPV of 100 %. Thus, p16 overexpression could be un-related to HPV oncogenic role in PC in at least 8,3 % of cases, also considering that such cases did not show HPV related morphology.

In this perspective, as in other district, the multimodal approach based on p16 IHC and HPV detection by molecular assay in PCs leads to a better identification of HPV-related subgroup, reducing the rate of both false-negative and false-positive cases [8, 12, 24, 30]. In consideration of the prognostic and predictive value of HPV infection, the choice of an accurate diagnostic assay for HPV detection is critical to define the most optimal management and follow-up for PC patients.

## Conclusions

Multiplex assay in the clinical practice could help to define the HPV-related PC subgroup avoiding the misclassification of these tumors. Our data confirm a high NPV of p16 intense overexpression, but a lower PPV, 8,3 % being the cases overexpressing p16 in absence of HPV-RNA. Thus, as p16 is a parameter potentially, but not exclusively, associated to HPV oncogenic activity, the multiplex approach let a simultaneous study of the HPV presence and its activity, differentiating the HPV-related from HPV-unrelated PCs. In conclusion, the multiplex HPV RNA ISH /p16 IHC assay could solve the diagnosis of HPV, especially in cases with discordant results. This approach has several advantages including the identification of the transcriptionally-active HPV and p16 on the same slide using a single technical approach, high performance in FFPE specimens, the complete not operator-dependent automation and the interpretation in the light field. Overall, the advantages of this new tool may allow wide spreadable for HPV routine testing in PC. Finally, our multiplex assay could help in the clinical practice the definition of HPV-related PC subset avoiding the misclassification of these tumors.

## Abbreviations

PC: Penile carcinoma
HPV: Human papillomavirus
HR-HPV: High-risk HPV
WHO: World Health Organization
PCR: Polymerase chain reaction
IHC: Immunohistochemistry
ISH: In situ hybridization
OPSCC: Oropharyngeal squamous cell carcinoma
Multiplex HPV RNA ISH /p16 IHC: Multiplex HPV RNA in situ hybridization/p16 Immunohistochemistry
FFPE: Formalin-fixed paraffin-embedded
DAB: Diaminobenzidine
USCC: Usual squamous cell carcinoma
PPV: Positive predictive value
NPV: Negative predictive value
