# Correction to: Discrepancy of p16 immunohistochemical expression and HPV RNA in penile cancer. A multiplex in situ hybridization/immunohistochemistry approach study

**DOI:** 10.1186/s13027-021-00363-6

**Published:** 2021-04-12

**Authors:** Federica Zito Marino, Rosalaura Sabetta, Francesca Pagliuca, Matteo Brunelli, Gabriella Aquino, Sisto Perdonà, Gerardo Botti, Gaetano Facchini, Francesco Fiorentino, Giovanni Di Lauro, Marco De Sio, Ferdinando De Vita, Giorgio Toni, Rodolfo Borges Dos Reis, Luciano Neder, Renato Franco

**Affiliations:** 1 Pathology Unit, Department of Mental and Physical Health and Preventive Medicine, University of Campania “L. Vanvitelli”, Complesso di Santa Patrizia, Via Luciano Armanni, 5, 80138 Naples, Italy; 2 Department of Pathology, University of Verona, Verona, Italy; 3 Pathology Unit, Istituto Nazionale Tumori, Fondazione G. Pascale, IRCCS, 80131 Naples, Italy; 4 Department of Urology, Istituto Nazionale Tumori, Fondazione G. Pascale, IRCCS, 80131 Naples, Italy; 5 Medical Oncology Unit, S.M. delle Grazie Hospital, Via Domitiana, 80078 Pozzuoli, NA Italy; 6 Pathology Unit, S.M. delle Grazie Hospital, Via Domitiana, 80078 Pozzuoli, NA Italy; 7 Urology Unit, S.M. delle Grazie Hospital, Via Domitiana, 80078 Pozzuoli, NA Italy; 8 Urology Unit, Department of Woman, Child and General and Specialized Surgery, University of Campania ’Luigi Vanvitelli, 80138 Naples, Italy; 9 Division of Medical Oncology, Department of Precision Medicine, School of Medicine, “Luigi Vanvitelli” University of Campania, Naples, Italy; 10 Laboratoire Central d’Anatomie pathologique, Hôpital universitaire de Nice, Université Côte d’Azur, 06000 Nice, France; 11 Department of Surgery and Anatomy, Urology Division, Ribeirao Preto School Medicine, University of São Paulo, Ribeirao Preto, 14049 900 Brazil; 12 Department of Pathology and Forensic Medicine, Ribeirão Preto Medical School, University of São Paulo, Ribeirão Preto, SP 14049 900 Brazil; 13 Molecular Oncology Research Center, Barretos Cancer Hospital, Barretos, SP 14784400 Brazil

**Correction to: Infect Agents Cancer 16, 22 (2021)**

**https://doi.org/10.1186/s13027-021-00361-8**

Following publication of the original article [1], the authors identified an error in the author name of Federica Zito Marino.

- The incorrect author name is: Federico Zito Marino
- The correct author name is: Federica Zito Marino

The author group has been updated above and the original article [1] has been corrected.
